# Concern and Risk Perception: Effects on Osteoprotective Behaviour

**DOI:** 10.1155/2014/142546

**Published:** 2014-09-08

**Authors:** A. L. Barcenilla-Wong, J. S. Chen, L. M. March

**Affiliations:** Institute of Bone and Joint Research, Kolling Institute of Medical Research, University of Sydney, Sydney, NSW 2065, Australia

## Abstract

This study aimed to determine the effect that level of concern for osteoporosis, as well as self-perceived risk of osteoporosis and fracture, has on supplementation use, seeking medical advice, bone mineral density (BMD) testing, and antiosteoporosis medication (AOM) use. Study subjects were 1,095 female Australian participants of the Global Longitudinal study of Osteoporosis in Women (GLOW) untreated for osteoporosis at baseline. Study outcomes from self-administered questionnaires included calcium and vitamin D supplementation, self-reported seeking of medical advice regarding osteoporosis, BMD testing, and AOM use in the last 12 months at the late assessment. Logistic regression was used in the analysis. Concern significantly increased the likelihood of seeking medical advice and, however, had no significant impact on screening or treatment. Heightened self-perceived risks of osteoporosis and fracture both significantly increased the likelihood of seeking medical advice and BMD testing while elevated self-perceived risk of fracture increased AOM use. Supplementation use was not significantly associated with concern levels and risk perception. Concern and risk perceptions to osteoporosis and fracture were significantly associated with certain bone-protective behaviours. However, the disconnect between perceived osteoporosis risk and AOM use illustrates the need to emphasize the connection between osteoporosis and fracture in future education programs.

## 1. Introduction

Osteoporosis is characterised by the reduction of bone mineral density (BMD) and the deterioration in bone architecture. Consequently, bone becomes fragile and is at increased risk of fracture. Osteoporosis affects both genders; however is more prevalent in women, particularly following menopause and has been reported to inflict a considerable amount of financial and personal burden. In Australia it has been reported that 22.8% of women aged ≥50 years had osteoporosis [1] with $1.9 billion being spent on direct costs associated with fractures [2]. Osteoporosis can inflict considerable affliction on individuals including decreased mobility, decreased quality of life, and increased risk of mortality following an osteoporotic fracture [3–5].

Osteoporosis is, however, preventable, and the magnitude of the burden it inflicts on individuals and societies alike can be reduced. Still, contrary to previous findings and recommendations elucidating the modifiable nature of osteoporosis through diet, exercise, supplementation, and medication [6, 7], these messages are oftentimes misplaced by individuals considering their own personal risk management. Previous studies have found that the lack of awareness of personal risk to osteoporosis resulted from a lack of knowledge about osteoporosis [8–10], but more alarmingly, the prevalent belief that osteoporosis is simply a consequential and unavoidable disease associated with ageing in older women [11].

Risk perception or perceived susceptibility has been previously described as an individual's subjective perception of the likelihood of developing a disease [13, 14]. It has been prominent in many theories, particularly when studying the adoption of health behaviours. The Health Belief model states that personal beliefs about a disease influence health behaviour and assume that high risk perceptions provide an impetus to adopting health-protective behaviours. Perceived susceptibility has been shown to be a strong predictor of preventive health behaviour second only to perceived barriers to adopting behaviour [13]. The role of emotions (including concern or worry) in decision-making has also been featured in health behaviour theories in recent times and has been shown to moderate or drive health-protective behaviours [15–17].

Little is known about the effect of concern and susceptibility on antiosteoporosis behaviour, particularly beyond cross-sectional analyses. Much can be gained from enhancing osteoporosis awareness and risk communication in the same way that understanding the dynamic between concern, perceived risk/susceptibility and behaviour has enhanced recommendations in education and prevention in breast cancer and coronary heart disease. However, studies have found that women are less “worried” or “concerned” about osteoporosis compared to other diseases such as cancer and cardiovascular disease [18–20]. It is therefore vital to understand the connection between bone health beliefs and bone protective behaviour in persons at risk of developing osteoporosis or with osteoporosis.

We hypothesise that a high level of concern as well as having a higher self-risk perception to osteoporosis and fracture are associated with individuals adapting behaviours in order to decrease their risk of osteoporosis and/or fracture. The purpose of this study is thus to determine the effect that level of concern for osteoporosis as well as self-perceived risk of osteoporosis and fracture has on antiosteoporosis behaviour such as (1) calcium and vitamin D supplementation, (2) seeking medical advice, (3) undergoing bone mineral (BMD) testing, and (4) taking antiosteoporosis medication (AOM).

## 2. Methods

### 2.1. GLOW Participants and Recruitment

The Global Longitudinal Study of Osteoporosis in Women (GLOW) is a longitudinal, observational cohort study of over 60,000 women ≥55 years from 17 study sites in 10 countries, including Australia. Details of the study were described previously [12, 21]. In Australia, a convenience sample of 8,029 eligible female patients was identified through 51 general practitioners from 14 practices around Sydney, between January 2007 and February 2008. General practitioners then mailed out a GLOW information packet containing study information, a participant consent form, and a reply-paid envelope to eligible females, inviting them to participate in the study [21]. Written consent was received from 3,011 (38%) patients who were then mailed the self-administered baseline GLOW questionnaire and a reply-paid envelope. Ninety-six percent (*n* = 2, 904) of patients (age ranging between 55 and 96 years) completed a baseline questionnaire and formed the Australian baseline GLOW study sample. Annual questionnaires were sent between 2007 and 2010 with a final follow-up response rate of 95%. The study was approved by the Northern Sydney Human Research Ethics Committee.

### 2.2. Study Subjects

Analyses were conducted on a subgroup of Australian GLOW participants and limited to women with at least two consecutive assessments who self-reported to never taking AOM. Women could contribute multiple observations if they had more than two assessments. In total, 1,095 women provided 2,874 observations for the study. Women included in the analysis were included if they provided information on concern for osteoporosis and self-perceived risk for osteoporosis and fracture at baseline and also at the earlier assessment for women who contributed multiple observations.

### 2.3. Questionnaires

Self-administered questionnaires exploring various aspects of bone health including patient characteristics, risk factors, use of medications, perception of risks, and health care use were sent annually from 2007–2010. Details regarding the questionnaire have been previously described [12]. “Concern about osteoporosis” (in thinking about your health, how concerned are you about osteoporosis?) was assessed by asking participants to rate their level of concern about osteoporosis using a 3-point Likert scale (i.e., very concerned, a little concerned, or not at all concerned). Perception of risk of getting osteoporosis (how would you rate your own risk of “getting osteoporosis” compared to other women your age?) and having a fracture (how would you rate your own risk of fracturing or breaking a bone compared to other women your age?) were both assessed by asking participants to rate their own risk of getting osteoporosis and also their own risk of having a fracture using a 5-point Likert scale (i.e., a lot lower, a little lower, about the same, a little higher, or much higher). Other information collected included age at the assessment, education level, private health insurance status, body mass index (BMI), weight lost in the previous year (≥5 kg), height lost since 25 years of age (>3 cm), smoking status, alcohol drinking, SF-36 physical function score and SF-36 vitality score using the Physical Functioning Scale of the 36-Item Short-Form Health Survey, European Quality of Life (EQ-5D) score, previous fracture since 45 years of age, history of parental hip fracture, maternal osteoporosis, comorbidities, and self-reported general health (i.e., excellent, very good, good, fair, and poor).

Study outcomes were self-reported use of supplements (i.e., calcium and/or vitamin D); self-reported seeking of medical advice regarding osteoporosis, self-reported BMD testing, and self-reported use of antiosteoporosis medications (i.e., estrogen, selective estrogen receptor modulators, bisphosphonates, calcitonin, parathyroid hormone, and strontium), in the last 12 months at their follow-up assessment.

### 2.4. Data Analysis

Descriptive statistics were used to describe baseline (first assessment) characteristics of Australian GLOW women who were included in this study. Cross-tabulation percentages were used to present relationships between concern about osteoporosis, perceived risk of osteoporosis and perceived risk of fractures at baseline. Logistic regression models were employed to determine the extent of associations of concern about osteoporosis, perceived risk of osteoporosis and perceived risk of fractures with the study outcomes at the next assessment (about 12 months later). The lack of independence in the study outcomes from multiple assessments in the same woman (clustering) was taken into account using generalised estimating equations (GEE).

## 3. Results

Data from 1,095 women who were not taking antiosteoporosis medication at baseline were included in the study. Details of respondents are shown in Table 1. The mean age of respondents was 66 years, of which 10.0% reported that they had been told by their doctor that they had osteoporosis (Table 1).

**Figure 1:**
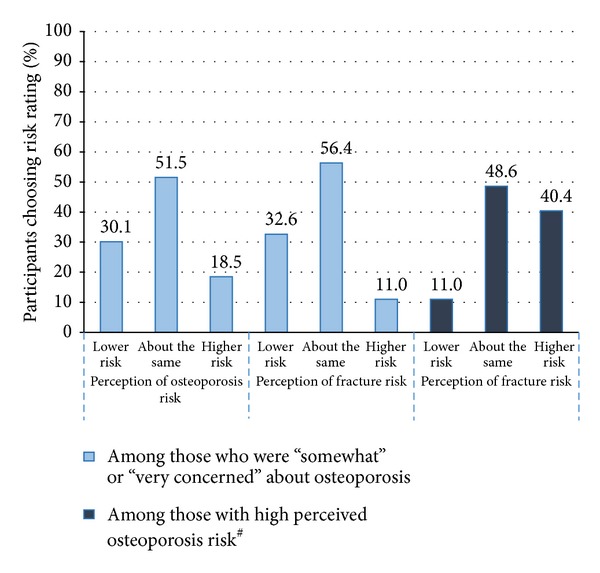
Percentage of risk perception to osteoporosis* and facture* by differing levels of concern about osteoporosis^∧^ and risk^#^. *Risk perception to osteoporosis (how would you rate your own risk of “getting osteoporosis” compared to other women your age?) and risk perception to fracture (how would you rate your own risk of fracturing or breaking a bone compared to other women your age?) were each assessed using 5-point Likert scales (i.e., much lower, a little lower, about the same, a little higher, or much higher) [12]. ^#^For this study: higher risk = “a little higher” or “much higher”. ^∧^Concern about osteoporosis (in thinking about your health, how concerned are you about osteoporosis?) was assessed using a 3-point Likert scale (i.e., very concerned, somewhat concerned, and not at all concerned) [12].

**Figure 2:**
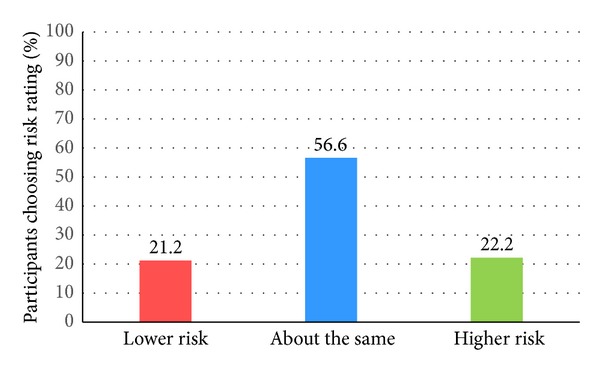
Percentage of perceived fracture risk* among untreated^∧^ respondents diagnosed^#^ with osteoporosis. *Risk perception to fracture (how would you rate your own risk of fracturing or breaking a bone compared to other women your age?) was each assessed using 5-point Likert scales (i.e., much lower, a little lower, about the same, a little higher, or much higher) [12]. For this study: lower risk = “much lower” or “a little lower”, about the same = “about the same,” and higher risk = “a little higher” or “much higher”. ^∧^Treatment was defined asself-reported use of antiosteoporosis medications (i.e., estrogen, selective estrogen receptor modulators, bisphosphonates, calcitonin, parathyroid hormone, and strontium).^ #^Self-reported osteoporosis (answer “yes” to “has a doctor or health provider ever told you that you had osteoporosis?”).

Figure 1 shows the percentage of baseline participant responses in relation to risk perception to osteoporosis and to fractures. The vast majority of respondents (81.6%) who were “somewhat” or “very concerned” about osteoporosis rated their risk of getting osteoporosis as being lower or the same as similar aged women. An even greater majority of women (89.0%) who were “somewhat” or “very concerned” about osteoporosis rated their risk of having a fracture as being lower or the same as similar aged women (Figure 1). Only a small percentage of respondents noted a higher self-risk rating to osteoporosis (18.5%) and fracture (11.0%). Of those who perceived themselves to have a higher risk to osteoporosis, only 40.4% noted a corresponding higher perceived risk to fracture.  Figure 2 shows that among those respondents who have been told that they had osteoporosis, only 22.2% rated their risk of fracture to be higher than other people their age despite not being on antiosteoporosis medication.

### 3.1. Effect of Concern and Risk Perception on Vitamin Supplementation Use

Table 2 depicts the association between the use of vitamin supplementation and concern and risk perception. Concern (*P* = 0.58), risk perception to osteoporosis (*P* = 0.13), and risk perception to fracture (*P* = 0.22) were not significantly associated with use of vitamin supplementation in the next 12 months (i.e., calcium and/or vitamin D) (Table 2).

### 3.2. Effect of Concern and Risk Perception on Seeking Medical Advice

Table 3 illustrates the association between concern and risk perception on seeking medical advice on osteoporosis (including things such as testing, treatment, and prevention). Concern was found to have a significant association to seeking medical advice (*P* < 0.001). The odds of seeking medical advice increased with increasing levels of concern for osteoporosis (“somewhat concerned”: OR 1.36; 95% CI: 1.08–1.71; “very concerned”: OR 2.11; 95% CI: 1.50–2.96). This significant association was also seen in risk perception to osteoporosis (*P* < 0.001) and fracture (*P* < 0.001). The odds of seeking medical advice increased with higher levels of risk perception to osteoporosis (“a little higher”: OR 3.13; 95% CI: 2.13–4.59; “much higher”: OR 2.71; 95% CI: 1.51–4.86) as well as with higher levels of risk perception to fracture (“a little higher”: 2.96; 95% CI: 1.94–4.52; “much higher”: OR 2.43; 95% CI: 1.17–5.06) (Table 3).

### 3.3. Effect of Concern and Risk Perception on Self-Reported Bone Mineral Testing (BMD)

Table 4 shows the association between concern and risk perception to BMD. There was a significant association between BMD testing and risk perception to osteoporosis (*P* = 0.03) as well as to risk perception to fractures (*P* = 0.03). The odds of having a BMD test increased with higher levels of risk perception to osteoporosis (“a little higher”: OR 2.13; 95% CI: 1.21–3.75; “much higher”: OR 2.53; 95% CI: 1.09–5.89) as well as with higher levels of risk perception to fracture (“a little higher”: 1.63; 95% CI: 0.85–3.14; “much higher”: OR 2.57; 95% CI: 0.86–7.63) (Table 4). Concern was not significantly associated with BMD testing in the subsequent follow-up assessment (*P* = 0.35) (Table 4).

### 3.4. Effect of Concern and Risk Perception on AOM

Table 5 presents the association between concern and risk perception to self-reported antiosteoporosis medication (AOM) use. The odds of taking AOM at the follow-up assessment were shown to increase, particularly with higher self-ratings of fracture risk (“a little higher”: OR: 1.99; 95% CI 0.80–4.92; “much higher”: OR 5.21; 95% CI 1.77–15.3) (Table 5). No significant association between concern and AOM use (*P* = 0.66) was seen, nor was AOM use associated with risk perception to osteoporosis (*P* = 0.06) (Table 5).

## 4. Discussion

The level of concern and perception of personal risk or susceptibility to a disease have been shown to modify an individual's behaviour in certain conditions but it have not been extensively studied in osteoporosis. In breast cancer, numerous studies have demonstrated a higher perception of risk to the disease increased adherence to mammography screening [22] and breast self-examination. A meta-analytical review of 19 studies between 1985 and 1993 found that those with higher vulnerability or felt susceptible to breast cancer were more likely to undergo mammography screening [23]. A later meta-analysis which combined the analysis completed by McCaul et al (1996) with 13 additional studies published between 1993–2002, suggested that perceived risk had a small but significant influence in mammography screening adherence [24]. A higher level of worry about breast cancer has also been found to be predictive of a greater likelihood for screening [23, 25]. In a similar way, higher perceptions of susceptibility to coronary heart disease (CHD) have also been found to be predictive of CHD preventive behaviours including, but not limited to, taking hormone replacement therapy (HRT), ingesting low fat, low cholesterol diets, and participating in exercise [26] as well as being less likely to smoke [27].

Concern and self-perceived risks to osteoporosis and future fracture have been found in this prospective study to affect certain behaviours such as seeking medical advice, BMD screening, and AOM in a prospective follow-up.

Concern as well as heightened self-perceived risks of osteoporosis and fracture significantly increased the likelihood of seeking medical advice. This confirms the hypothesis and concurs with findings by Campbell and Roland that perceived susceptibility was a key motivator to seeking medical care [28]. In the same study those who consulted their doctors the least generally were also less anxious about their health and were also less concerned about symptoms while those who consulted their doctors the most thought they were the most susceptible to disease [28]. Although these results are to be expected, this is the first time the effect of concern and risk perception on seeking medical advice has been explored in osteoporosis.

Heightened self-perceived risks of osteoporosis and fracture were both associated with BMD testing. Although the positive effect of risk perception on BMD testing has been previously described in cross-sectional analyses [29] and concurs with the current findings; the prospective effect on disease screening has not been previously studied in osteoporosis. The current findings also agree with those found in both prospective and cross-sectional studies concerning breast and colon cancers. A prospective study by Sutton et al. found that those who perceived themselves to be more at risk of breast cancer were more likely go for breast screening [30]. A cross-sectional study by Savage and Clarke (1996) found that the intention to have a mammogram in Australian women aged between 50 and 70 years was significantly associated to perceived susceptibility to breast cancer [31], while McCaul et al. found perceived susceptibility to be related to both mammography and breast self-examination [32]. In colon cancer, a prospective cohort study on male automotive workers found perceived susceptibility to improve weaker intentions to screen over time [33]. Cross-sectional studies on colon cancer also found greater odds of screening associated with higher risk perceptions to colon cancer [34] with one study purporting that a higher colon cancer risk perception produced 1.7 greater odds of having a past colonoscopy [35].

An elevated self-perceived risk of fracture was also found to increase the likelihood of taking AOM. This concurs with findings from a cross-sectional analysis of a US cohort of postmenopausal women where higher perceived risk of fracture was related to significantly higher AOM treatment rates compared to those with the same/lower risk of fracture [36]. Although these are results that can be expected this is the first time the perceived risks were seen to affect behaviours prospectively.

In Australia, information about the benefits of bone-related supplementation is frequently featured in prominent mass media campaigns. The ubiquitous nature of this information and accessibility of “over-the-counter” supplementation lead to the common general usage of calcium and vitamin D to prevent osteoporosis amongst many other conditions. In the current study, calcium and/or vitamin D use is relatively high with 51% of respondents being on some form of supplementation. When taking risk perception status into consideration, supplementation is varied but still remains high with 48% of those who had both a low perception of osteoporosis risk and a low perception of fracture risk and 65% of those with both a high perception of osteoporosis and a high perception of fracture risk being on some form of supplementation. The high general usage in the current sample may explain why concern and perception were not associated with the use of supplementation.

An interesting finding that emerged in the current study was the apparent disconnect between concern, perception of risk, and behaviour. Concern was not found to be significantly associated with behaviour besides seeking medical advice. The majority of participants were concerned about osteoporosis with 71% (775/1,086) being either “very” or “somewhat” concerned about the disease. However, participants did not necessarily connect this concern to their own risk perception to developing osteoporosis or having a future fracture. The level of concern was also not shown to affect bone protective behaviour to reduce these risks (such as screening and treatment). Among women who were either “somewhat” or “very concerned” about osteoporosis at baseline, 30% (228/758) rated their risk of getting osteoporosis to be lower than other women their age. A similar pattern was seen in fracture risk perception with 33% (249/763) of those concerned about osteoporosis perceiving their risk of future fracture as being lower compared to similar aged women.

Secondly, as being osteoporotic increases your risk to fracture it can be expected that if you perceive yourself to be at high risk of getting osteoporosis, you would also perceive yourself to be at a higher risk of fracture. It can also be expected that if you consider yourself to be at high risk of a disease, one will take actions to reduce these risks (and in terms of the current study, this consists of taking AOM). The majority of this sample of postmenopausal women reported having a “lower or same” risk compared to other women their age rating their own risk of getting osteoporosis (82%) and future fracture (89%). Of the women with a high risk perception of getting osteoporosis, only 40% also rated their risk of fractures as being higher than other women their age (Figure 1). Further, of those women who had reported being told by their doctor that they had osteoporosis, only 22% rated their risk of fracture to be higher than similar aged women despite not being on any AOM (Figure 2). This illustrated that the majority of women failed to associate the link between future fracture risk with having a high risk of getting osteoporosis, with the link also missing despite having a diagnosis of osteoporosis.

This lack of association between osteoporosis and fractures concurs with findings from a study by Giangregorio et al., where 54% of women who had suffered a fragility fracture failed to attribute their fracture to osteoporosis [37]. In the current study, the disconnect between osteoporosis and fractures was also shown to have a follow through effect on antiosteoporosis behaviour with only a higher risk perception of having a fracture being associated with treatment, while risk perception to getting osteoporosis had no significant effect. Although, these findings are contrary to those expected, they do concur with previous studies where perception alone did not dictate protective behaviours. Phillipov et al. found in a cross-sectional study that Australian women who had a high risk perception to osteoporosis were 1.6 times less likely to adapt any preventative action towards osteoporosis [38], while Chang et al. found that despite 54% of women believing that they were at risk for osteoporosis, 64% of these women perceived barriers to taking actions to reduce this risk [39].

The findings of the study indicate a need to explore the reasons behind this disconnection in a more qualitative way and a need to target certain groups particularly those at risk of having osteoporosis who do not perceive their own risk to osteoporosis and fracture. Linking osteoporosis firmly to risk of fractures should also be the focus of future osteoporosis education programs as it was only risk perception to fractures and not risk perceptions to osteoporosis that was associated with osteoporosis pharmacologic therapy. Appreciating the link between osteoporosis and fractures is vital and has been described previously by Beaton et al. as the “aha” moment that provides the impetus for patients to move towards positive bone-protective actions such as BMD testing and AOM, if required [40].

This study has limitations that need to be considered before interpreting the findings. Participants of the study were recruited as a convenient sample predominantly from regions of Sydney that may be considered to be within a higher socioeconomic background with the majority of the sample attaining at least a Higher School Certificate (12 years of study) as well as having private health insurance cover. The level of education in the current sample (77%) is contrasted to the 65% of those attaining a similar level of education in the greater Sydney area. Previous studies have linked a higher educational attainment to a greater knowledge of osteoporosis while others found greater knowledge to affect protective behaviours. The majority of women in the current sample (95%) also reported having private health insurance. This may explain the high supplementation rate found in this sample as these women would be more likely to afford supplementation. By taking these into consideration, the perception and behaviours of participants used in the current study could have possibly been influenced by their socioeconomic background. Future studies may need to be conducted in a more diverse sample population.

The authors recognise that the decision-making process involved in behaviour is one which is multifactorial. By the very nature of the study, it was difficult to recruit people who have “never” had a bone density test prior to entering the study as it was found that 74% answered “yes” to having a BMD test. Likewise, 57% (624) of those included in the current analysis answered “yes” to ever having a BMD test at baseline. Of these, 90 (14%) were validated by locating bone density results directly from patients and/or physician records with 5 considered osteoporotic (*T*-Score ≤ −2.5) and 27 considered osteopenic (−1 ≤* T*-Score ≤ −2.5). The proportion of those included in the analysis that may have had previous exposure to BMD and knowledge of their results may have influenced behaviour.

Although, the quantitative nature of the surveys which forms the basis of the current analyses does not allow for the extrapolation of reasons behind perception and action, it does, however, identify that there are indeed gaps in association of risk as well as barriers present when considering certain protective behaviours to osteoporosis in this group of women. For future studies, a more qualitative approach is needed to overcome the barriers and further enhance self-efficacy in bone-protective behaviours such as BMD screening and treatment compliance.

Concern and risk perceptions to osteoporosis and fracture have been found to be significantly associated with certain bone-protective behaviours in this prospective study. However, the apparent lack of association between concern and risk perception as well as between perceived risk of osteoporosis and fracture illustrates the need for future studies to explore this disconnect further and also challenges future education programs to emphasize the connection between osteoporosis and fracture.

## Figures and Tables

**Table 1:** Baseline (first assessment) characteristics of the study women.

	1,095 women from Australian GLOW cohort
Age (years), mean (standard deviation)	66 (9.4)
Body mass index (kg/m^2^), number (%)	
<18.5	20 (2)
18.5–24.9	429 (43)
25.0–29.9	318 (32)
≥30	221 (22)
Private health insurance, number (%)	1035 (95)
Prior year weight loss (≥5 kgs), number (%)	84 (8)
Height lost since 25 years old (>3 cm), number (%)	187 (21)
Current Smoking, number (%)	65 (6)
Alcohol drinking (≥7 drinks/week), number (%)	354 (32)
GH (general health) score, number (%)	
Excellent	161 (15)
Very good	430 (39)
Good	366 (32)
Fair	120 (11)
Poor	18 (2)
SF-36 physical function score∗, mean (standard deviation)	77 (24.1)
SF-36 vitality score∗, mean (standard deviation)	62 (19.2)
EQ-5D score^∧^, mean (standard deviation)	0.81 (0.20)
Maternal osteoporosis, number (%)	173 (22)
Parental hip fracture, number (%)	171 (16)
Prior fracture after 45 years, number (%)	194 (18)
Ever diagnosed with, number (%)	
Asthma	162 (15)
Chronic bronchitis or emphysema	53 (5)
High cholesterol	559 (52)
Hypertension	513 (48)
Heart disease	108 (10)
Osteoporosis	102 (10)
Osteoarthritis/degenerative joint disease	351 (33)
Rheumatoid arthritis	77 (7)
Education level, number (%)	
School certificate (year 11 or less)	365 (34)
Higher school certificate	160 (15)
Trade certificate I, II, III, or IV	60 (6)
Diploma or advanced diploma	164 (15)
Bachelor degree	116 (11)
Graduate certificate/graduate degree	121 (11)
Higher degree (masters or doctorate)	89 (3)

*According to the Physical Functioning Scale of the 36-Item Short-Form Health Survey.

^∧^European Quality of Life (EQ-5D) score.

**Table 2:** Odds ratio (ORs) of calcium and/or vitamin D use in the next 12 months for covariates about concern and risk perception of osteoporosis (limited to assessments with noncurrent use of calcium or vitamin D).

	Number (%)	Unadjusted OR (95% CI)	*P* ^∧^	Adjusted OR∗ (95% CI)	*P* ^∧^
Concern about osteoporosis^#^ (using a 3-point Likert scale)			0.30		0.58
Not at all concerned	128 (22.3)	1.00		1.00	
Somewhat concerned	203 (23.4)	1.05 (0.82–1.36)		1.02 (0.77–1.36)	
Very concerned	32 (28.1)	1.34 (0.85–2.11)		1.21 (0.72–2.04)	
Perception of osteoporosis risk^#^ (using a 5-point Likert scale)			0.04		0.13
Much lower	70 (20.3)	1.00		1.00	
A little lower	85 (23.2)	1.17 (0.82–1.67)		1.03 (0.69–1.53)	
About the same	150 (22.1)	1.10 (0.80–1.52)		1.10 (0.76–1.58)	
A little higher	47 (38.2)	2.42 (1.54–3.79)		2.47 (1.45–4.18)	
Much higher	5 (16.7)	0.81 (0.30–2.15)		0.30 (0.07–1.33)	
Perception of fracture risk^#^ (using a 5-point Likert scale)			0.24		0.22
Much lower	77 (22.1)	1.00		1.00	
A little lower	83 (22.1)	1.00 (0.70–1.42)		0.99 (0.67–1.45)	
About the same	170 (23.5)	1.08 (0.80–1.48)		1.12 (0.78–1.59)	
A little higher	26 (31.3)	1.61 (0.95–2.73)		1.93 (1.06–3.51)	
Much higher	4 (21.1)	0.95 (0.31–2.93)		0.17 (0.02–1.34)	

Note: Each assessment was treated as an observation, and lack of independence between assessments for the same women (clustering) was taken into account using generalized estimating equations.

^#^Concern about osteoporosis (In thinking about your health, how concerned are you about osteoporosis?); perception of osteoporosis risk (How would you rate your own risk of “getting osteoporosis” compared to other women your age?); and perception of fracture risk (How would you rate your own risk of fracturing or breaking a bone compared to other women your age?).

∗Adjusted for age, body mass index, private health insurance status, level of education, smoking, drinking, fracture since age 45 years, a maternal history of osteoporosis, history of fractured hip among parents, height loss since age 25 years (≥3 cms), weight loss (≥5 kgs) in the last year, self-reported health status, SF-36 physical score, seeking medical advice on osteoporosis in the previous year and any prior bone mineral density testing.

^∧^Test for trend by treating groups as an ordered (continuous) variable.

**Table 3:** Odds ratio (ORs) of seeking medical advice in the next 12 months for covariates about concern and risk perception of osteoporosis.

	Number (%)	Unadjusted OR (95% CI)	*P* ^∧^	Adjusted OR∗ (95% CI)	*P* ^∧^
Concern about osteoporosis^#^ (using a 3-point Likert scale)			<0.001		<0.001
Not at all concerned	160 (17.7)	1.00		1.00	
Somewhat concerned	457 (28.0)	1.56 (1.26–1.91)		1.36 (1.08–1.71)	
Very concerned	123 (39.9)	2.48 (1.84–3.35)		2.11 (1.50–2.96)	
Perception of osteoporosis risk^#^ (using a 5-point Likert scale)			<0.001		<0.001
Much lower	80 (14.9)	1.00		1.00	
A little lower	169 (25.5)	1.67 (1.24–2.23)		1.54 (1.13–2.11)	
About the same	310 (25.3)	1.72 (1.30–2.26)		1.61 (1.19–2.19)	
A little higher	136 (43.2)	3.36 (2.39–4.73)		3.13 (2.13–4.59)	
Much higher	41 (46.7)	3.45 (2.09–5.69)		2.71 (1.51–4.86)	
Perception of fracture risk^#^ (using a 5-point Likert scale)			<0.001		<0.001
Much lower	87 (15.5)	1.00		1.00	
A little lower	196 (27.3)	1.79 (1.35–2.36)		1.60 (1.18–2.17)	
About the same	344 (26.4)	1.69 (1.29–2.21)		1.66 (1.23–2.24)	
A little higher	93 (44.9)	3.20 (2.21–4.62)		2.96 (1.94–4.52)	
Much higher	24 (48.0)	3.60 (1.94–6.68)		2.43 (1.17–5.06)	

Note: Each assessment was treated as an observation and lack of independence between assessments for the same women (clustering) was taken into account using generalized estimating equations.

^#^Concern about osteoporosis (in thinking about your health, how concerned are you about osteoporosis?); perception of osteoporosis risk (how would you rate your own risk of “getting osteoporosis” compared to other women your age?); and perception of fracture risk (how would you rate your own risk of fracturing or breaking a bone compared to other women your age?).

∗Adjusted for age, body mass index, private health insurance status, level of education, smoking, drinking, fracture since age 45 years, a maternal history of osteoporosis, history of fractured hip among parents, height loss since age 25 years (≥3 cms), weight loss (≥5 kgs) in the last year, self-reported health status, SF-36 physical score, seeking medical advice on osteoporosis in the previous year, and any prior bone mineral density testing.

^∧^Test for trend by treating groups as an ordered (continuous) variable.

**Table 4:** Odds ratio (ORs) of bone mineral density (BMD) testing in the next 12 months for covariates about concern and risk perception of osteoporosis (limited to assessments with non-BMD testing in the previous year^!^).

	Number (%)	Unadjusted OR (95% CI)	*P* ^∧^	Adjusted OR∗ (95% CI)	*P* ^∧^
Concern about osteoporosis^#^ (using a 3-point Likert scale)			0.002		0.35
Not at all concerned	86 (12.5)	1.00		1.00	
Somewhat concerned	168 (15.8)	1.31 (0.99–1.73)		1.10 (0.80–1.51)	
Very concerned	38 (21.7)	1.93 (1.26–2.96)		1.28 (0.77–2.13)	
Perception of osteoporosis risk^#^ (using a 5-point Likert scale)			0.001		0.03
Much lower	39 (9.9)	1.00		1.00	
A little lower	78 (17.1)	1.87 (1.24–2.83)		1.75 (1.121–2.73)	
About the same	122 (14.6)	1.55 (1.06–2.28)		1.40 (0.91–2.15)	
A little higher	36 (20.9)	2.41 (1.47–3.96)		2.13 (1.21–3.75)	
Much higher	12 (25.0)	2.99 (1.44–6.23)		2.53 (1.09–5.89)	
Perception of fracture risk^#^ (using a 5-point Likert scale)			0.004		0.03
Much lower	40 (9.9)	1.00		1.00	
A little lower	85 (16.7)	1.83 (1.23–2.73)		1.59 (1.02–2.48)	
About the same	139 (16.3)	1.78 (1.23–2.60)		1.70 (1.11–2.61)	
A little higher	22 (17.7)	1.95 (1.11–3.43)		1.63 (0.85–3.14)	
Much higher	6 (23.1)	2.63 (0.99–6.96)		2.57 (0.86–7.63)	

Note: Each assessment was treated as an observation and lack of independence between assessments for the same women (clustering) was taken into account using generalized estimating equations.

^#^Concern about osteoporosis (in thinking about your health, how concerned are you about osteoporosis?); perception of osteoporosis risk (how would you rate your own risk of “getting osteoporosis” compared to other women your age?); and perception of fracture risk (how would you rate your own risk of fracturing or breaking a bone compared to other women your age?).

∗Adjusted for age, body mass index, private health insurance status, level of education, smoking, drinking, fracture since age 45 years, a maternal history of osteoporosis, history of fractured hip among parents, height loss since age 25 years (≥3 cms), weight loss (≥5 kgs) in the last year, self-reported health status, SF-36 physical score, current use of calcium and/or vitamin D, and seeking medical advice on osteoporosis in the previous year.

^∧^Test for trend by treating groups as an ordered (continuous) variable.

^!^Australian government provides subsided BMD testing once every two years for people at high fracture risk (e.g., prior fragility fracture, aged >70 years, and long-term glucocorticoid users).

**Table 5:** Odds ratio (ORs) of anti-osteoporosis medication (AOM) use in the next 12 months for covariates about concern and risk perception of osteoporosis.

	No. (%)	Unadjusted OR (95% CI)	*P* ^∧^	Adjusted OR∗ (95% CI)	*P* ^∧^
Concern about osteoporosis^#^ (using a 3-point Likert scale)			0.08		0.66
Not at all concerned	33 (3.6)	1.00		1.00	
Somewhat concerned	64 (3.9)	1.08 (0.71–1.66)		0.88 (0.54–1.44)	
Very concerned	20 (6.5)	1.84 (1.04–3.27)		1.28 (0.65–2.54)	
Perception of osteoporosis risk^#^ (using a 5-point Likert scale)			0.001		0.06
Much lower	13 (2.4)	1.00		1.00	
A little lower	22 (3.3)	1.39 (0.69–2.78)		1.33 (0.64–2.76)	
About the same	55 (4.5)	1.90 (1.03–3.52)		1.66 (0.85–3.23)	
A little higher	16 (5.1)	2.17 (1.03–4.58)		1.87 (0.81–4.35)	
Much higher	9 (10.5)	4.74 (1.96–11.5)		2.48 (0.82–7.51)	
Perception of fracture risk^#^ (using a 5-point Likert scale)			0.24		0.002
Much lower	14 (2.5)	1.00		1.00	
A little lower	20 (2.8)	1.11 (0.56–2.22)		0.95 (0.46–1.96)	
About the same	60 (4.6)	1.88 (1.04–3.39)		1.77 (0.93–3.37)	
A little higher	15 (7.3)	3.05 (1.45–6.43)		1.99 (0.80–4.92)	
Much higher	8 (16.0)	7.43 (2.95–18.7)		5.21 (1.77–15.3)	

Note: Each assessment was treated as an observation, and lack of independence between assessments for the same women (clustering) was taken into account using generalized estimating equations.

^#^Concern about osteoporosis (In thinking about your health, how concerned are you about osteoporosis?); perception of osteoporosis risk (How would you rate your own risk of “getting osteoporosis” compared to other women your age?); and perception of fracture risk (How would you rate your own risk of fracturing or breaking a bone compared to other women your age?).

∗Adjusted for age, body mass index, private health insurance status, level of education, smoking, drinking, fracture since age 45 years, a maternal history of osteoporosis, history of fractured hip among parents, height loss since age 25 years (≥3 cms), weight loss (≥5 kgs) in the last year, self-reported health status, SF-36 physical score, current use of calcium and/or vitamin D, seeking medical advice on osteoporosis in the previous year and any prior bone mineral density testing.

^∧^Test for trend by treating groups as an ordered (continuous) variable.
